# Hierarchical Carbon Micro/Nanonetwork with Superior Electrocatalysis for High‐Rate and Endurable Vanadium Redox Flow Batteries

**DOI:** 10.1002/advs.201801281

**Published:** 2018-10-31

**Authors:** Wei Ling, Qi Deng, Qiang Ma, Hong‐Rui Wang, Chun‐Jiao Zhou, Jian‐Kai Xu, Ya‐Xia Yin, Xiong‐Wei Wu, Xian‐Xiang Zeng, Yu‐Guo Guo

**Affiliations:** ^1^ College of Science Hunan Agricultural University Changsha Hunan 410128 P. R. China; ^2^ CAS Key Laboratory of Molecular Nanostructure and Nanotechnology CAS Research/Education Center for Excellence in Molecular Sciences Institute of Chemistry Chinese Academy of Sciences (CAS) Beijing 100190 P. R. China; ^3^ Hunan Province Yin Feng New Energy Co. Ltd. Changsha Hunan 410000 P. R. China

**Keywords:** carbon electrodes, durable cycle life, hierarchical carbon micro/nanonetworks, high‐rate performance, vanadium redox flow batteries

## Abstract

Vanadium redox flow batteries (VRFBs) are receiving increasing interest in energy storage fields because of their safety and versatility. However, the electrocatalytic activity of the electrode is a pivotal factor that still restricts the power and cycling capabilities of VRFBs. Here, a hierarchical carbon micro/nanonetwork (HCN) electrode codoped with nitrogen and phosphorus is prepared for application in VRFBs by cross‐linking polymerization of aniline and physic acid, and subsequent pyrolysis on graphite felt. Due to the hierarchical electron pathways and abundant heteroatom active sites, the HCN exhibits superior electrocatalysis toward the vanadium redox couples and imparts the VRFBs with an outstanding energy efficiency and extraordinary stability after 2000 cycles at 250 mA cm^−2^ and a discharge capacity of 10.5 mA h mL^−1^ at an extra‐large current density of 400 mA cm^−2^. Such a micro/nanostructure design will force the advancement of durable and high‐power VRFBs and other electrochemical energy storage devices.

With the ever‐increasing advancement of sustainable development strategies, the utilization of clean and renewable energy sources tends to replace fossil fuels.^1^, ^2^ However, these sustainable energy, such as solar and wind energies, need large‐scale energy storage systems to stabilize the energy output and achieve their practical applications.^3^, ^4^, ^5^ Among these systems, the vanadium redox flow battery (VRFB) shows important advantages of a flexible design, long cycle life, and low cost of maintenance.^6^, ^7^, ^8^ Actually, VRFB employs the same element with different valence state as positive and negative reaction species, which largely restrains the problems of cross‐contamination between positive and negative electrolyte compared with other redox flow batteries. And as the redox reaction zone, the electrode material greatly determines the activation and concentration polarization of the redox reaction and has an enormous impact on the energy efficiency (EE) and capacity of VRFBs.^9^, ^10^, ^11^ Therefore, the development of electrode materials with a suitable design is imperative for enhancing the overall performance of VRFBs.

Because of their low cost and excellent acid resistance, carbon‐based materials (CMs) have been widely used as electrodes for VRFBs. However, the small interfacial area and inadequate active sites of CMs result in poor electrocatalytic activity toward redox reactions and largely hinder the comprehensive performance of VRFBs. Two methods have been adopted to enhance the electrocatalytic performance of VRFB electrodes. One is to improve the catalytic activity through surface oxygen functionalization,^12^, ^13^, ^14^ metal^15^ and metal oxide^16^, ^17^, ^18^, ^19^ deposition, and defect site^20^, ^21^, ^22^, ^23^, ^24^ and heteroatom doping,^25^, ^26^, ^27^, ^28^, ^29^, ^30^, ^31^, ^32^, ^33^, ^34^ largely boosting the conductivity and charge transfer capability of the electrodes.^35^, ^36^ The other is to increase the amount of active sites with the main focus of increasing the contact area by constructing multidimensional structures using carbon nanotubes,^37^, ^38^, ^39^, ^40^ graphene oxide,^41^, ^42^, ^43^, ^44^ porous carbon,^45^, ^46^ and other functional materials.^47^, ^48^ This strategy has also aroused substantial attention in the VRFB field, however, the rate capability and cycling stability of the electrodes need to be further improved to satisfy the requirements of the high power and stable output of VRFBs in the future. Currently, achieving this goal with one electrode remains a challenge. Recently, the micro/nanostructured materials have showed great merits among electrochemical energy storage systems,^49^, ^50^, ^51^ such as rechargeable batteries, fuel cells, and supercapacitor. The micromaterials play the part of macroskeleton to provide electronic transportation and distribution or protect the overall structure, while the nanomaterials equipped a large zone for charge transfer and mass diffusion due to high specific surface area. Therefore, the integrated micro/nanostructured structure possesses the comprehensive advantages of the functional materials as micrometer and nanometer scales, which can effectively boost the rapid transportation and uniform distribution of electrons and ions in the electrode.

Herein, we prepare a hierarchical carbon micro/nanonetwork (HCN) consisting of a graphite felt (GF) fibers with micronetwork and a carbon nanonetwork on the GF via crosslinking polymerization of aniline and phytic acid and subsequent pyrolysis. The HCN structure vastly increases the electrode/electrolyte interfacial area and facilitates charge transfer and mass transfer for the vanadium redox reactions. Such a structure provides plentiful electron transport channels, which significantly improve electron transport and distribution and serve as a flexible but robust skeleton for the vanadium electrolyte to flow through. Furthermore, the codoped nitrogen and phosphorus introduced from aniline and phytic acid create abundant catalytically active sites in the carbon nanonetwork and improve the charge transfer capacity of the electrode. As a result, the VRFB based on the HCN possesses an outstanding EE and extraordinary stability after over 2000 cycles at 250 mA cm^−2^ and reveals a high capacity of 10.5 mA h mL^−1^ at a particularly large current density of 400 mA cm^−2^.

The fabrication of the HCN is schematically illustrated in **Figure** **1**. First, the aniline monomers crosslink and polymerize on the GF with phytic acid serving as the crosslinking agent. After hydrothermal and carbonization reaction, an HCN with nitrogen and phosphorus codopants is formed. Based on scanning electron microscopy (SEM) characterization, the smooth surface of the pristine GF (**Figure** **2**a,d) becomes rough in the HCN (Figure 2b,e) after crosslinking polymerization. The rough surface is clearly covered by a nanofiber network (Figure 2b). After ultrasonic processing for 2 h, nanofibers with 40 nm in diameter separate from the HCN, and the obtained nanofibers have an amorphous structure based on examination by transmission electron microscopy (TEM) (Figure 2c). Furthermore, the nanofibers consist of carbon, oxygen, nitrogen, and phosphorus (Figure S1, Supporting Information) uniformly distributed on the GF, as shown by energy‐dispersive spectroscopy (EDS) (Figure 2g). Note that phytic acid is essential for triggering the formation of such a micro/nanostructure. Undergoing the polymerization and carbonization of aniline on GF fibers (CA‐GF) without adding phytic acid, only carbon granules randomly distribute on the GF (Figure S2c, Supporting Information), by contrast, in the presence of phytic acid, a nanocarbon network forms on the GF surface. However, Figure S2a,b (Supporting Information) reveals that PA‐GF fabricated by an identical process for HCN without aniline possesses a similar smooth surface as GF, which explains why phytic acid alone cannot form the nanofiber network on GF. The above results indicate that phytic acid contributes to the formation and stabilization of the carbon nanonetwork on the HCN.

**Figure 1:**
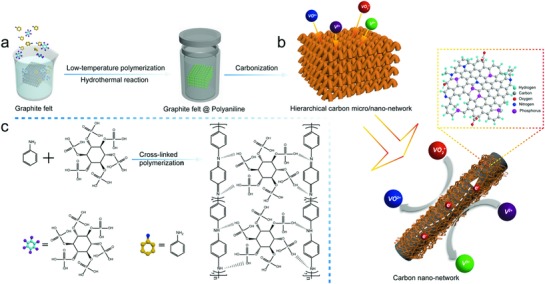
Schematic diagram of the preparation process for the HCN. a) The low‐temperature polymerization of aniline monomers occurs on the GF with phytic acid as the crosslinking agent following a hydrothermal reaction; b) through a carbonization process, nitrogen and phosphorus codopants are introduced into the crosslinking carbon nanonetwork formed on the surface of GF; c) the equation corresponding to the crosslinking polymerization reaction.

**Figure 2:**
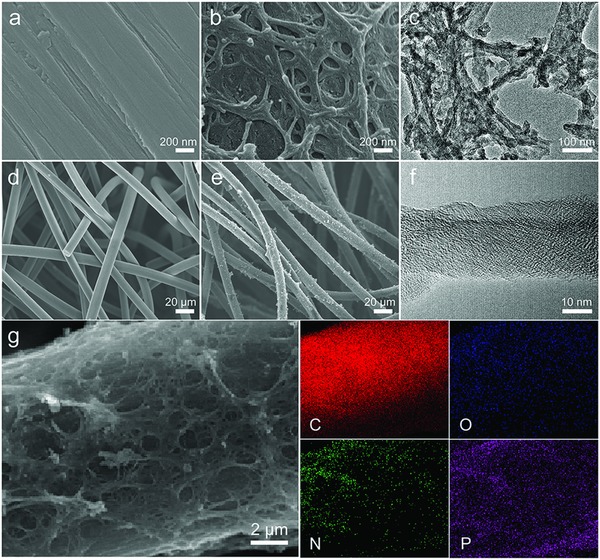
Morphological characterization of the HCN by electron microscopy. SEM images of a,d) GF and b,e) the HCN at different magnifications; c,f) TEM image of the HCN; and g) elemental mapping images of C, O, N, and P for the HCN.

The lattice defects of GF, CA‐GF, and the HCN were detected by X‐ray diffraction (XRD). All the diffraction patterns show two peaks at 26.5° and 44.0°, attributed to the (002) and (100) planes, respectively, of the graphite lattice (**Figure** **3**a). GF and CA‐GF show similar diffraction peak shapes, while the HCN possesses two wider and weaker diffraction peaks, suggesting that the HCN possesses a larger number of defect active sites. The structural properties of the obtained materials were analyzed with the help of Raman spectroscopy (Figure 3b). The Raman signals observed at 1584 cm^−1^ (G band) and 1340 cm^−1^ (D band) are related to the in‐plane vibrations of sp^2^ carbon atoms and disordered structures, respectively, and the defect level of the carbon materials can be evaluated in terms of the intensity ratio of the G and D bands (*I*_D_/*I*_G_). Obviously, the *I*_D_/*I*_G_ (1.01) ratio of the HCN is higher than that of GF (0.98) and CA‐GF (0.97), implying that a larger number of exposed graphitic defects are present in the HCN. The improved defect level of HCN is mainly attributed to heteroatom doping of the crosslinked carbon nanonetwork, which can serve as redox‐active sites for the vanadium redox reactions.

**Figure 3:**
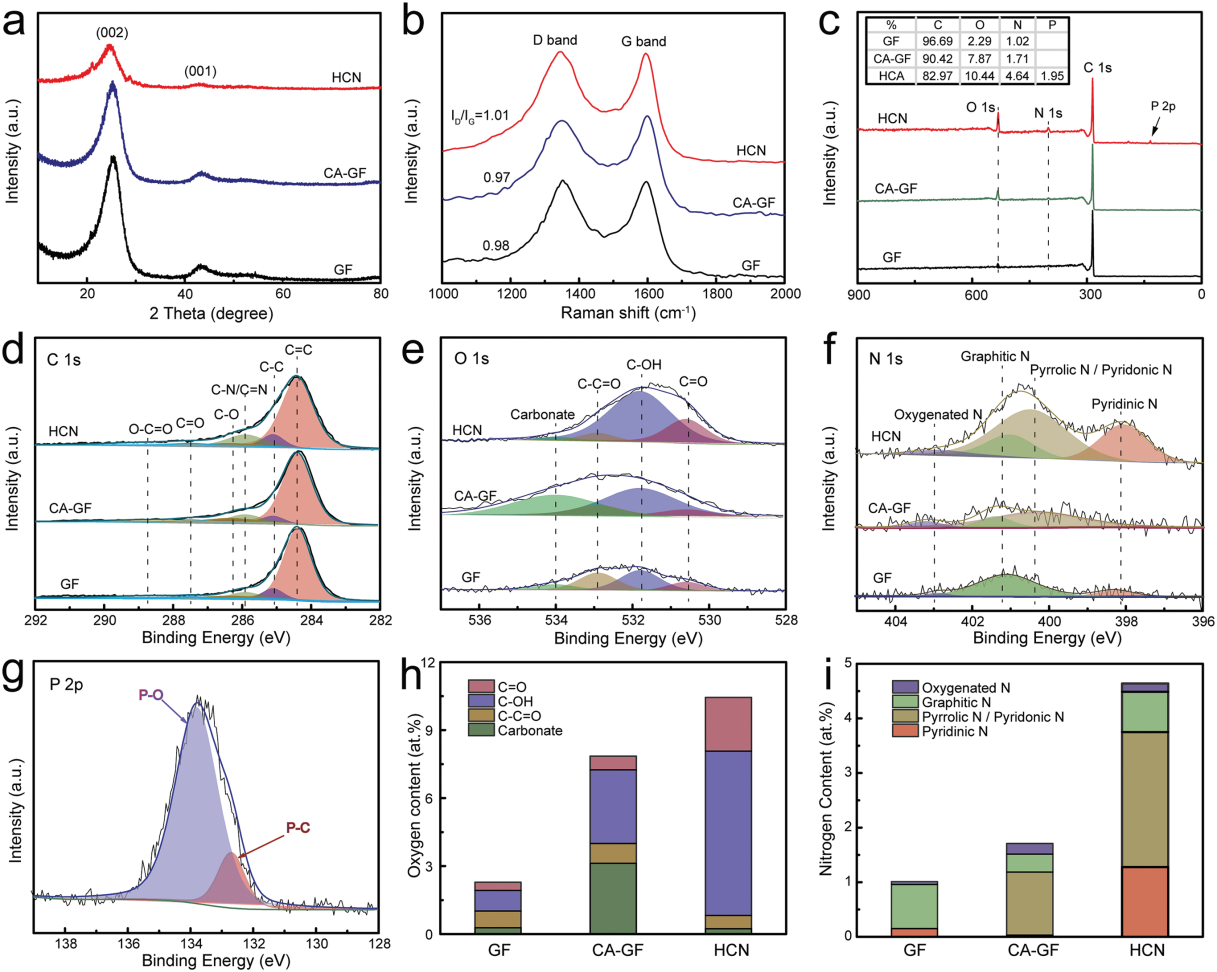
Composition and structural analysis of the HCN by spectroscopy. a) XRD pattern. b) Raman spectra. c) XPS survey scans with various element contents and fitting of the high‐resolution d) C 1s, e) O 1s, and f) N 1s spectra of GF, CA‐GF, and the HCN. g) High‐resolution XPS P 2p spectrum of HCN. The relative contents of h) oxygen and i) nitrogen functional groups in GF, CA‐GF, and the HCN.

Further, the compositions and relative contents of all materials were analyzed by X‐ray photoelectron spectroscopy (XPS). The XPS spectra of CA‐GF and GF present three characteristic peaks corresponding to C 1s, O 1s, and N 1s, while the HCN exhibits an additional characteristic peak attributed to P 2p (Figure 3c), illustrating that phosphorus atoms were successfully doped in the HCN. Moreover, the nitrogen and oxygen contents of these electrodes follow the order HCN > CA‐GF > GF, which indicates that the HCN contains the most electrocatalytically active sites toward vanadium redox reactions. And C 1s spectra were deconvoluted into six peaks (Figure 3d) corresponding to O—C=O (288.7 eV), C=O (287.5 eV), C—O (286.3 eV), C—N/C=N (285.9), C—C (285.1 eV), and C=C units (284.4 eV),^52^ and the O 1s spectra (Figure 3e) consisted of four peaks attributed to C=O (530. 6), C—OH (531.8), C—C=O (532.9), and carbonate groups (534 eV).^27^, ^53^ The contents of the various oxygen‐containing functional groups are compared in Figure 3h. Due to the hydrogen bond effect among amino and water molecules in the processes of the polymerization and hydrothermal reaction, the CA‐GF possesses an improved oxygen content after carbonization reaction.^54^ However, the oxygen contents of the HCN largely surpass those of GF and CA‐GF, especially for the hydroxyl groups, which can serve as efficient active sites that promote the redox reactions of the vanadium species, suggesting that the HCN possesses on excellent electrocatalytic capacity. The peaks in the N 1s region (Figure 3f) are assigned to pyridinic N (398.2 eV), pyrrolic or pyridonic N (400.3 eV), graphitic N (401.0 eV), and oxygenated N (402.5 eV),^55^, ^56^ and their relative amounts are shown in Figure 3i. The nitrogen species contents of the HCN are remarkably higher than those of GF and CA‐GF, particularly for pyridinic N and pyrrolic N, which better enhance the catalytic activity toward the vanadium redox reactions than the other nitrogen catalytic species due to the existence of a lone pair of electrons. The high nitrogen content of the HCN possesses a lower energy barrier for the vanadium redox reactions.^41^ Moreover, phosphorus was successfully doped in the HCN, and its signal was fitted as P—O (133.8 eV) and P—C (132.7) (Figure 3g).^57^ Due to the differences in size and electronegativity, the P doping can break the stability of hexatomic carbon ring with P—π bond, and induce uneven distribution of charge surrounding carbon atoms in the pristine carbon‐based materials,^25^ then the heteroatom doping can give rise to the formation of the defect and vacancy on the carbon electrode surface, which can elevate the electrical conductivity and charge transfer capacity. According to some recent reports, the N—O codoping has the synergistic catalytic effect on the vanadium redox reactions.^58^, ^59^, ^60^ In this work, the P element, as another dopant, can not only improve the electrochemical property of the electrode, but also increase the content of oxygen functional groups deriving from the existence of P—O bond. Therefore, due to the synergistic catalytic effect of N, O, and P elemental codoping, the HCN possesses abundant catalytically active sites, which will significantly enhance the electrocatalytic capacity toward the vanadium redox reactions on the HCN electrode.

Cyclic voltammetry (CV) curves were used to evaluate the electrocatalytic performance of the various electrodes (**Figure** **4**). The peak current ratio of the oxidation and reduction processes (*I*_pa_/*I*_pc_) shows the reversibility of the vanadium redox reaction, and the peak potential difference (∆*E* = *E*_pa_ − *E*_pc_) represents the polarization of the electrochemical process. For the positive reaction (Figure 4a), the *I*_pa_/*I*_pc_ ratio for the HCN (0.95) is much closer to 1 than that of GF (0.70) and CA‐GF (0.91), implying that the HCN possesses excellent reversibility in the positive electrode reaction. In addition, the △*E* (349 mV) value of the HCN is approximately half that of GF (670 mV), suggesting a dramatic decrease in the polarization of the VO^2+^/VO_2_^+^ redox couple. Likewise, the HCN also possesses outstanding electrocatalytic activity for the V^2+^/V^3+^ redox couple on the negative side (Figure 4b). In addition, HCN processes larger effective areas toward vanadium ions reaction based on the calculation of the electrochemical surface area (ECSA),^58^, ^59^ and corresponding data are collected (Table S1, Supporting Information). The prominent electrocatalytic properties of the HCN are ascribed to the large interfacial area and abundance of catalytically active sites for the vanadium redox reactions. By contrast, PA‐GF and GF exhibit similar inferior CV behaviors (Figure S3, Supporting Information), indicating that phytic acid alone cannot effectively improve the electrochemical performance of GF. Thus, the HCN doped with heteroatoms indeed plays a significant role in improving the electrocatalytic performance.

**Figure 4:**
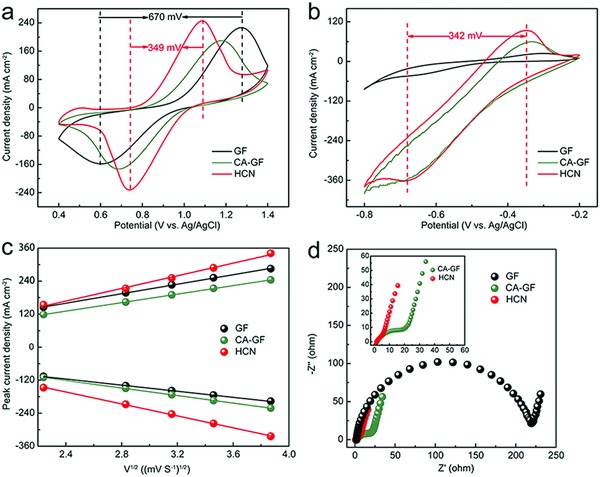
Electrochemical performance measurements. a) The positive and b) negative CV curves of the GF, CA‐GF, and HCN in 3 mol L^−1^ H_2_SO_4_ solution containing 0.1 mol L^−1^ VOSO_4_. c) Plots of the oxidation and reduction peak current density versus the square root of scan rate for GF, CA‐GF, and HCN. d) Nyquist plots of the GF, CA‐GF, and HCN in 3 mol L^−1^ H_2_SO_4_ solution containing 0.1 mol L^−1^ VOSO_4_.

Additionally, CV tests were performed at different scan rates (Figure S4a–c, Supporting Information). Compared with the shapes of the oxidation and reduction peaks for GF and CA‐GF, those for the HCN exhibit less variation with increasing scan rate, indicating that the HCN exhibits better electrochemical stability. Moreover, the *I*_pa_/*I*_pc_ ratios of the HCN are all much closer to 1 than those of GF and CA‐GF at different scan rates (Figure S4d, Supporting Information), indicating that the HCN possesses outstanding reversibility toward the VO^2+^/VO_2_^+^ redox reaction. In addition, the diffusion process is known to control the electrochemical reaction of the VO^2+^/VO_2_^+^ couple based on the good linear relationship of the peak current density versus square root of scan rates (Figure 4c).^61^ In addition, the diffusion rate can be estimated from the following Randles–Sevcik equation^62^

(1) $$i_{p}=2.99\times 10^{5}\alpha^{1/2}An^{3/2}D_{0}{}^{1/2}v^{1/2}C_{0}$$

where *i*_p_ represents the peak current of the oxidation or reduction process, α expresses the charge transfer coefficient, *A* is the effective area, *n* is the number of electrons transferred during the electrochemical reaction, *v* is the scan rate, *D*_0_ is the diffusion coefficient, and *C*_0_ is the concentration of vanadium ions. Therefore, the diffusion rates of the vanadium species on the electrode can be evaluated from the slope of the line in the plot of the peak current against the square root of the scan rate. As shown in Figure S5 (Supporting Information), the slopes of the oxidation and reduction processes on the HCN are, respectively, 114.8 and 108.7, which are much larger than those of GF (86.2, 55.4) and CA‐GF (77.4, 68.5). Therefore, the HCN exhibits outstanding vanadium ion diffusion, which is ascribed to the fact that the HCN structure provides rapid transport pathways and homogeneous electron and ion distributions in the electrode.

The charge transfer resistance (*R*_ct_) during the redox reaction was measured by electrochemical impedance spectroscopy (EIS) (Figure 4d). The semicircle in the high‐frequency region is attributed to the *R*_ct_, and the sloped line in the low‐frequency region arises from the mass diffusion resistance through the electrolyte.^23^ The *R*_ct_ value of the HCN is ≈2.58 Ω, which is substantially lower than those of GF (208.30 Ω) and CA‐GF (10.14 Ω), and the equivalent circuit is shown in Figure S6 (Supporting Information). The prominent charge transfer capability of the composite electrode is ascribed to the fact that the HCN structure dramatically enhances the interfacial area between the electrode and electrolyte and that heteroatom doping introduces an abundance of redox‐active sites for the vanadium redox reactions.

The GF, CA‐GF, and HCN were assembled to evaluate their effectiveness on VRFB performance (**Figure** **5** and Figure S7, Supporting Information). Compared with GF and CA‐GF, the HCN exhibits a lower charging plateau and a higher discharging plateau, and the corresponding overpotential is decreased by 225 mV, implying a small polarization effect on the charge–discharge process. The discharge capacity of the HCN‐based VRFB (24.1 mA h mL^−1^) is 104.3% higher than that of the GF‐based VRFB (11.8 mA h mL^−1^) at a current density of 250 mA cm^−2^ (Figure 5a). Even at 400 mA cm^−2^, the discharge capacity of the HCN‐based VRFB still reaches 10.5 mA h mL^−1^, indicating the excellent rate capacity of the electrode (Figure 5b). The small polarization effect and excellent rate capacity arise from the HCN structure, which enhances the utilization of the vanadium electrolyte and facilitates the rapid transfer of ions and electrons in the electrode. The columbic efficiency (CE) of the various electrodes exhibits less discrepancy in the range from 100 to 400 mA cm^−2^ (Figure 5c and Figure S7c, Supporting Information), while the voltage efficiency (VE) of the HCN‐based VRFB is higher than those of the CA‐GF/GF‐based VRFBs, indicating that the HCN possesses a low polarization effect toward the electrochemical reaction. The EE of the HCN‐based VRFB is 12% higher than that of the GF‐based VRFB at 250 mA cm^−2^ (Figure 5d). Prominently, the HCN enables the VRFB to work steadily at 400 mA cm^−2^ with an EE of 56.2%, while the VRFBs based on the other electrode materials function at current densities below 350 mA cm^−2^ (Figure S7d, Supporting Information), a detailed comparison of these VRFBs is provided in Table S2 (Supporting Information). Accordingly, the VRFB based on the HCN possesses an outstanding cycling stability at 250 mA cm^−2^ with an EE of 70.4% without obvious decay after 2000 charge–discharge cycles, while the GF‐based VRFB fails to function beyond 400 cycles. The excellent cycling performance of the VRFB based on the HCN surpasses that in most reported works (**Figure** **6**). Such a comprehensively outstanding outcome is ascribed to the fact that the HCN structure provides sufficient effective contact area for ion and electron transfer between the electrode and electrolyte and abundant catalytically active sites on the HCN, vastly promoting the electrochemical activity toward the vanadium redox reactions during long‐term operation.

**Figure 5:**
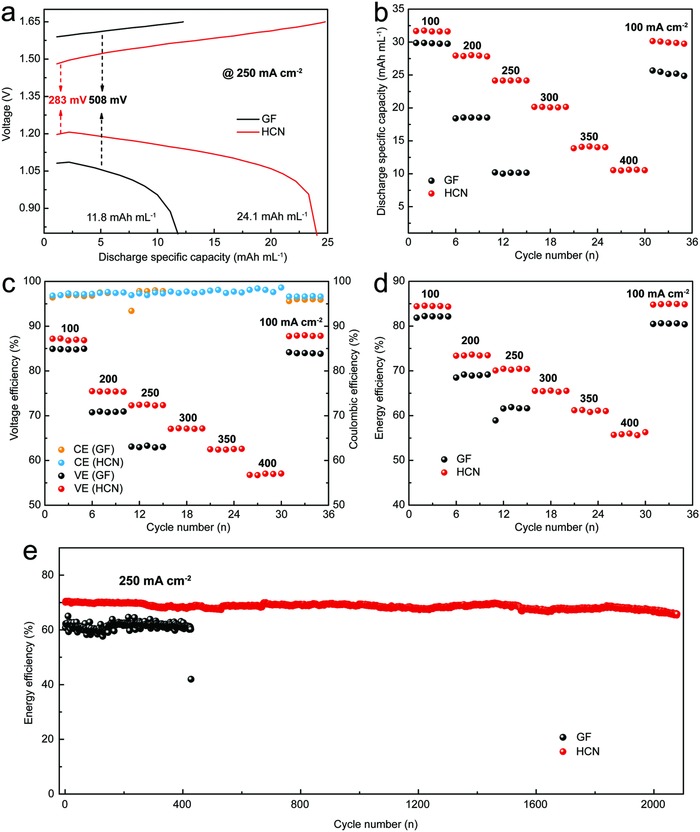
Performance tests for VRFBs. a) Charge–discharge curves of the GF and HCN at 250 mA cm^−2^, b) discharge specific capacity, c) CE and VE, d) EE of GF and HCN at different current densities, and e) cycling performances of VRFBs based on GF and HCN electrodes at 250 mA cm^−2^.

**Figure 6:**
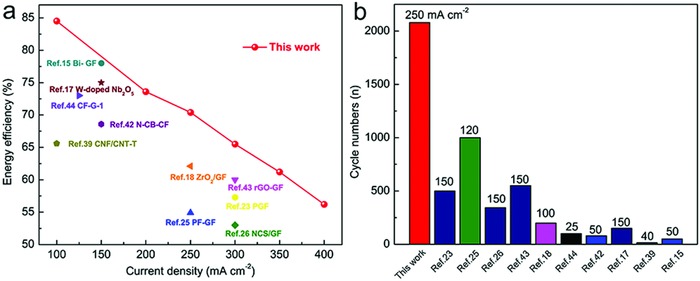
VRFB performance comparison with previous reports. Comparative evaluation of a) EE and b) lifespan of VRFBs among recently reported works.

In this work, we developed an HCN material doped with heteroatoms via a two‐stage process of crosslinking polymerization and carbonization. The HCN structure provides rapid transport and a uniform distribution of electrons and ions in the electrode, and the abundant heteroatom active sites give rise to excellent electrocatalytic activity toward the vanadium redox reactions, thereby imparting the VRFB with a comprehensively superior performance. It is reasonable to believe that this work will advance the development of durable and high‐power VRFBs and other electrochemical energy storage devices.

## Experimental Section

*Preparation of the HCN Electrode*: The HCN electrode was fabricated via a simple two‐step process of crosslinking polymerization and carbonization. First, 50 mL of a phytic acid solution (6%) and 5 mL of aniline monomer were thoroughly mixed, and GFs (3 cm × 4 cm) were immersed in the above solution under stirring. Next, 1.92 g of ammonium persulfate, used as an initiator, was dissolved into 10 mL of deionized water. The two solutions were mixed together after cooling to ≈4 °C and continuously stirred for 12 h. The resulting GFs and pulpy products were transferred to a Teflon‐lined autoclave and heated at 100 °C for 5 h. The obtained GFs were washed with deionized water and dried at 100 °C for 24 h. Then, the GFs were carbonized in a quartz tube furnace at 900 °C for 2 h under an argon atmosphere. Finally, the prepared GF was washed several times with deionized water and alcohol and dried at 100 °C for 10 h. The obtained GF electrode was labeled HCN. GF composite electrodes named CA‐GF and PA‐GF were fabricated by an identical process as the HCN without the addition of phytic acid or aniline, respectively.

*Structural Characterization*: The morphologies of the various materials were recorded via SEM (SU8020) operated at 10 kV and TEM (JEM‐2100F) operated at 200 kV. The defect and graphitization levels of the samples were determined by XRD (D/max 2500), and Raman spectra were collected with a 532 nm laser (Lab RAM HR Evolution). The element contents and compositions of the samples were determined by XPS (ESCALAB250XI) using a Thermo Scientific ESCA Lab 250Xi instrument with 200 W Al Kα radiation and a base pressure of 3 × 10^−10^ mbar in the analysis chamber.

*Electrochemical Measurements*: CV was performed on a CHI760D electrochemical workstation. Platinum and silver chloride (Ag/AgCl) electrodes were used as the counter and reference electrodes, respectively, and the composite electrodes served as the working electrode (0.5 cm × 0.5 cm). The electrolyte consisted of 0.1 mol L^−1^ VOSO_4_ in 3 mol L^−1^ H_2_SO_4_. EIS measurements were conducted with a 5 mV amplitude in a frequency range of 0.01–100 kHz at the open‐circuit potential, and the data were fitted with Z‐view software. VRFB single batteries using GF, CA‐GF, or the HCN (2 × 2 cm) as both positive and negative electrodes were assembled for charge–discharge tests conducted with a battery test system (Land CT2001A). The electrolyte consisted of 0.75 mol L^−1^ VOSO_4_ + 0.375 mol L^−1^ V_2_ (SO_4_)_3_ in 3 mol L^−1^ H_2_SO_4_ (15 mL) on both sides with Nafion 115 as a proton exchange membrane. The voltage windows of the discharge and charge measurements were, respectively, set as 1.55–0.95 V at 100 and 200 mA cm^−2^ and as 1.65–0.8 V at large current densities from 250 to 400 mA cm^−2^.

## Conflict of Interest

The authors declare no conflict of interest.

## Supporting information

Supplementary
