# Supplementary material for: Hierarchical Carbon Micro/Nanonetwork with Superior Electrocatalysis for High‐Rate and Endurable Vanadium Redox Flow Batteries
Source: Adv Sci (Weinh). 2018 Oct 31;5(12):1801281. doi: 10.1002/advs.201801281 (PMC6299713; doi:10.1002/advs.201801281)
Supplement: Supplementary file 1 — Supplementary [file ADVS-5-1801281-s001.pdf]

Copyright WILEY-VCH Verlag GmbH & Co. KGaA, 69469 Weinheim, Germany, 2018.

ADVANCED SCIENCE Open Access

Supporting Information

for *Adv. Sci.,* DOI: 10.1002/advs.201801281

Hierarchical Carbon Micro/Nanonetwork with Superior Electrocatalysis for High-Rate and Endurable Vanadium Redox Flow Batteries

Wei Ling, Qi Deng, Qiang Ma, Hong-Rui Wang, Chun-Jiao Zhou, Jian-Kai Xu, Ya-Xia Yin, Xiong-Wei Wu, Xian-Xiang Zeng,* and Yu-Guo Guo**

Copyright WILEY-VCH Verlag GmbH & Co. KGaA, 69469 Weinheim, Germany, 2016.

Supporting Information

Hierarchical Carbon Micro/nano-network with Superior Electrocatalysis for High-rate and Endurable Vanadium Redox Flow Batteries

Wei Ling†, Qi Deng†, Qiang Ma, Hong-Rui Wang, Chun-Jiao Zhou, Jian-Kai Xu, Ya-Xia Yin, Xiong-Wei Wu, Xian-Xiang Zeng*, and Yu-Guo Guo**

W. Ling, Q. Deng, Q. Ma, H-R Wang, Dr. C.-J. Zhou, Dr. J.-K. Xu, Dr. X.-W. Wu, Dr. X.-X. Zeng
College of Science, Hunan Agricultural University, Changsha, Hunan 410128, P. R. China
E-mail: wxwcsu05@aliyun.com, xxzeng@hunau.edu.cn

W. Ling, Q. Ma, Dr. Y.-X. Yin, Prof. Y.-G. Guo
CAS Key Laboratory of Molecular Nanostructure and Nanotechnology, CAS Research/Education Center for Excellence in Molecular Sciences, Institute of Chemistry, Chinese Academy of Sciences (CAS), Beijing 100190, P. R. China
E-mail: ygguo@iccas.ac.cn

Q. Deng, Dr. X.-W. Wu
Hunan Province Yin Feng New Energy Co. LTD, Changsha, Hunan 410000, P. R. China

† These authors contributed equally to this work.

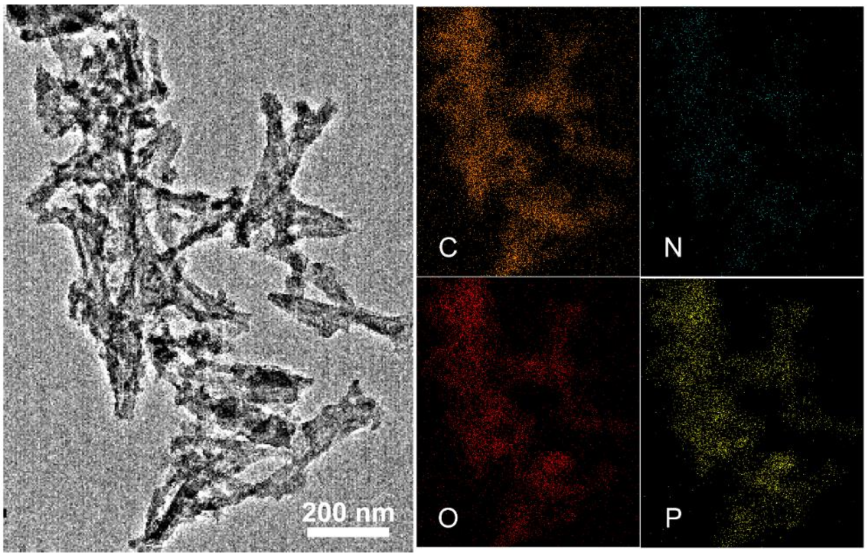

Figure S1. TEM image and corresponding EDS mappings of the nanofiber network. The structure of the nanofiber network and its elements distribution of C, N, O and P.

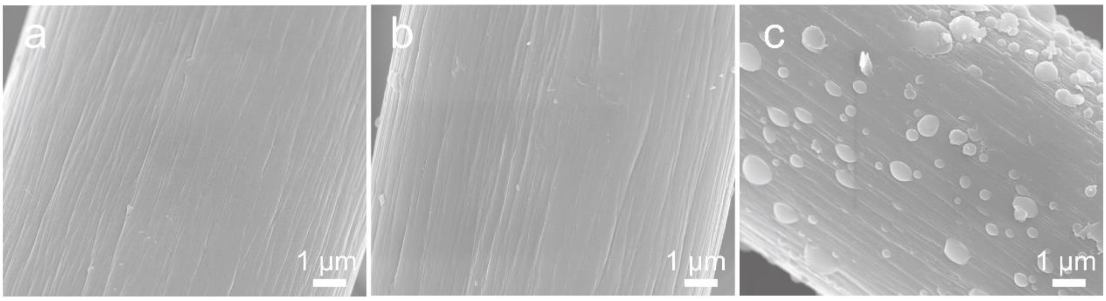

Figure S2. SEM images of different electrode materials. (a) GF, (b) PA- GF and (c) CA-GF.

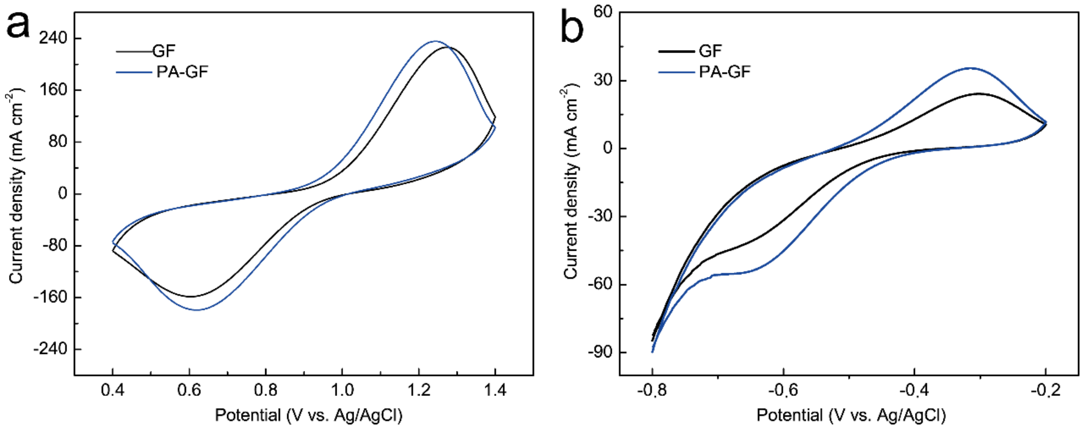

Figure S3. CV curves comparison of the GF and PA-GF. (a) Positive and (b) negative CV curves in 3 mol L^{-1} H_2SO_4 solution containing 0.1 mol L^{-1} $VOSO_4$.

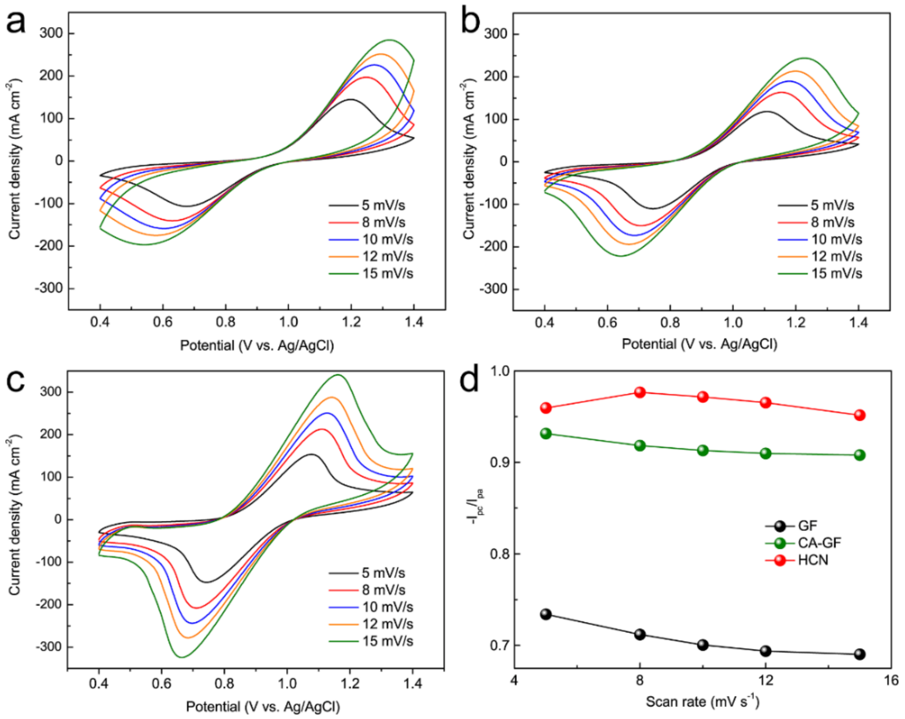

Figure S4. CV curves of various electrode at various scan rates. (a) GF, (b) CA-GF, (c) HCN, and (d) corresponding I_{pc}/I_{pa} values versus different scan rates in 3 mol L^{-1} H_2SO_4 solution containing 0.1 mol L^{-1} $VOSO_4$.

Table S1. The CV data comparison of GF, CA-GF and HCN.

Positive half-cell

electrode	I_{pc}	I_{pa}	V_{pa}	V_{pc}	$-I_{pc}/I_{pa}$	ΔE	ECSA
	(mA cm^{-2})	(mA cm^{-2})	(V)	(V)		(mV)	(m^2)
GF	-158.56	226.36	1.272	0.605	0.70	667	1.04×10^{-6}
CA-GF	-173.16	189.68	1.177	0.688	0.91	489	1.14×10^{-6}
HCN	-232.36	245.44	1.086	0.739	***0.95***	***347***	1.52×10^{-6}

Negative half-cell

electrode	I_{pc}	I_{pa}	V_{pa}	V_{pc}	$-I_{pc}/I_{pa}$	ΔE	ECSA
	(mA cm^{-2})	(mA cm^{-2})	(V)	(V)		(mV)	(m^2)
GF	-	24.1	-0.305	-	-	-	1.58×10^{-7}
CA-GF	-376.08	59.64	-0.332	-0.750	6.31	418	3.91×10^{-7}
HCN	-361.96	93.76	-0.348	-0.690	***3.86***	***342***	6.15×10^{-7}

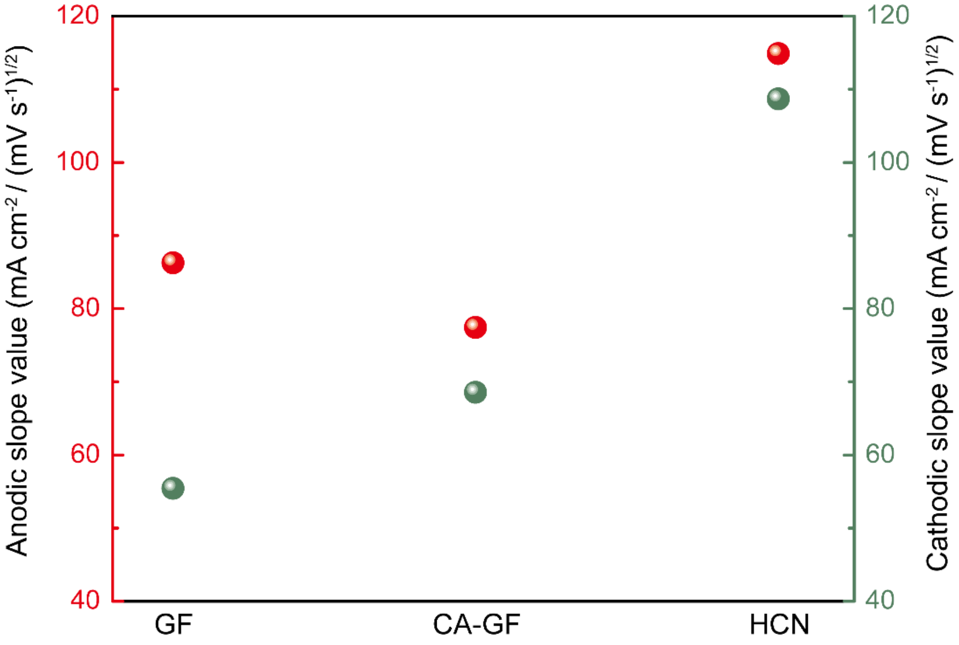

Figure S5. The diffusion capacity assessment of vanadium ions. Slope value of peak current density versus square root of scan rates for GF, CA-GF and HCN.

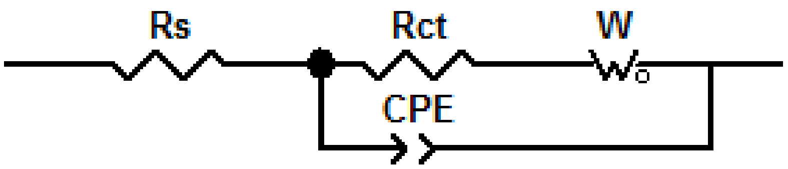

Figure S6. The equivalent circuit representing the circuit elements in the Nyquist spectra. The equivalent circuit is shown in Figure S3. R_s represents the solution resistance, contact resistance and electrode resistance. R_{ct} stands for the electron transfer resistance across the electrode/solution interface, the double-layer capacitance, and W is the Warburg impedance.

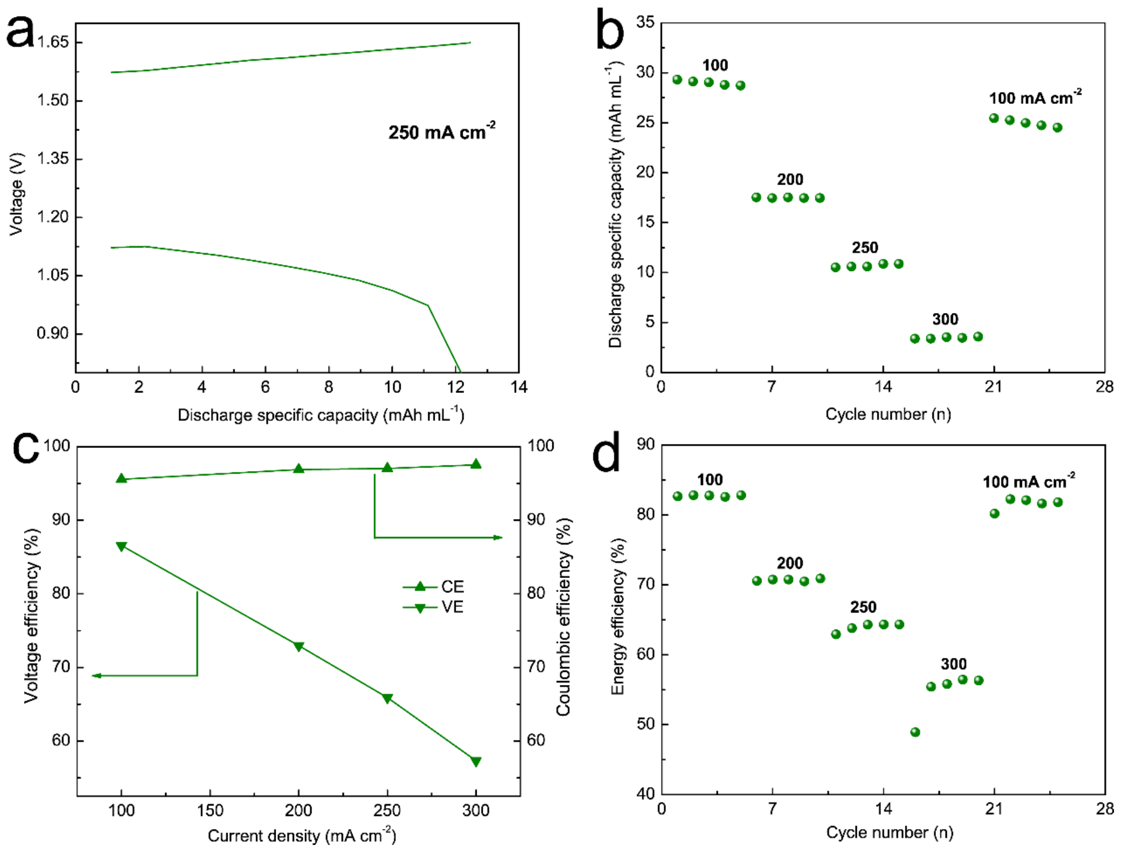

Figure S7. Electrochemical performance characterization analysis. (a) Charge-discharge curves of the CA-GF at 250 mA·cm^{-2}, (b) Discharge specific capacity, (c) EE, (d) CE and VE of the CA-GF at various current density.

Table S2. The cycle efficiency data of GF, CA-GF and HCN.

Sample	Efficiency	Current density (mA cm^{-2})					
		100	200	250	300	350	400
GF	EE	81.8	68.4	61.5			
	VE	84.8	70.7	62.9			
CA-GF	EE	82.6	70.6	64.0	55.4		
	VE	86.5	72.9	65.8	56.8		
HCN	EE	84.5	73.6	70.4	65.5	61.2	56.2
	VE	87.1	75.4	72.4	67.1	62.4	57.0
